# *C*-type cytochrome-initiated reduction of bacterial lytic polysaccharide monooxygenases

**DOI:** 10.1042/BCJ20210376

**Published:** 2021-07-28

**Authors:** Jessie Branch, Badri S. Rajagopal, Alessandro Paradisi, Nick Yates, Peter J. Lindley, Jake Smith, Kristian Hollingsworth, W. Bruce Turnbull, Bernard Henrissat, Alison Parkin, Alan Berry, Glyn R. Hemsworth

**Affiliations:** 1 Astbury Centre for Structural Molecular Biology and School of Molecular and Cellular Biology, Faculty of Biological Sciences, University of Leeds, Leeds LS2 9JT, U.K.; 2 Department of Chemistry, University of York, York YO10 5DD, U.K.; 3 School of Chemistry and Astbury Centre for Structural Molecular Biology, University of Leeds, Leeds, U.K.; 4 Architecture et Fonction des Macromolécules Biologiques (AFMB), CNRS, Aix-Marseille Université, Marseille, France; 5 AFMB, INRA, USC 1408, Marseille, France; 6 Department of Biological Sciences, King Abdulaziz University, Jeddah, Saudi Arabia

**Keywords:** biofuels, cytochrome, electron transfer, LPMO, oxidation–reduction, protein structure

## Abstract

The release of glucose from lignocellulosic waste for subsequent fermentation into biofuels holds promise for securing humankind's future energy needs. The discovery of a set of copper-dependent enzymes known as lytic polysaccharide monooxygenases (LPMOs) has galvanised new research in this area. LPMOs act by oxidatively introducing chain breaks into cellulose and other polysaccharides, boosting the ability of cellulases to act on the substrate. Although several proteins have been implicated as electron sources in fungal LPMO biochemistry, no equivalent bacterial LPMO electron donors have been previously identified, although the proteins Cbp2D and E from *Cellvibrio japonicus* have been implicated as potential candidates. Here we analyse a small *c*-type cytochrome (*Cj*X183) present in *Cellvibrio japonicus* Cbp2D, and show that it can initiate bacterial Cu^II/I^ LPMO reduction and also activate LPMO-catalyzed cellulose-degradation. In the absence of cellulose, *Cj*X183-driven reduction of the LPMO results in less H_2_O_2_ production from O_2_, and correspondingly less oxidative damage to the enzyme than when ascorbate is used as the reducing agent. Significantly, using *Cj*X183 as the activator maintained similar cellulase boosting levels relative to the use of an equivalent amount of ascorbate. Our results therefore add further evidence to the impact that the choice of electron source can have on LPMO action. Furthermore, the study of Cbp2D and other similar proteins may yet reveal new insight into the redox processes governing polysaccharide degradation in bacteria.

## Introduction

The production of second-generation biofuels from lignocellulosic waste in a cost-effective manner is a much-sought-after goal of the industrial biotechnology sector [1,2]. One of the major hurdles to overcome has been the inherent difficulty in liberating individual glucose units from cellulose, a polymer of β-1,4-glucose, for subsequent fermentation into bioethanol. This is a difficult obstacle to overcome because the polysaccharide substrate is highly recalcitrant. As such, efforts to use cellulose as a feedstock for industrial bioethanol production often faltered [3]. However, the discovery of lytic polysaccharide monooxygenases (LPMOs) has caused considerable renewed interest in this area because LPMOs boost the activity of cellulase cocktails, making cellulose deconstruction significantly more cost effective [4–9].

LPMOs are widespread and have been discovered in all kingdoms of life. Based on their protein sequences, these enzymes are classified into seven distinct families in the CAZy database of carbohydrate-active enzymes (www.cazy.org) — auxiliary activity (AA) families AA9 to AA11 and AA13 to AA16 [6–17]. Much of the research on LPMOs has focussed on understanding how these enzymes bring about the observed boosting of cellulase activity. The molecular basis for this is thought to result from their ability to introduce chain breaks into the crystalline regions of cellulose, thereby introducing new points at which cellulases can act ([7,9,18–21] and see [22] for a recent review). LPMOs do this by reductively activating oxygen or hydrogen peroxide to specifically attack at either the C1 or C4 carbon of the glucose ring resulting in hydroxylation at that position and subsequent breakage of the glycosidic bond [7,9,18–20,23]. The precise mechanism of reaction that these enzymes use is a hotly debated topic, which continues to fuel much of the research on these enzymes at present (see [22,24,25] for recent reviews).

Though LPMO families share little sequence identity, they are united by the presence of the ‘histidine brace’ motif [7,26–29]. This motif coordinates a single copper ion in the active site which is essential for activity. Probing how this mononuclear metal site can activate dioxygen has been the key driver behind a number of recent mechanistic studies [18,27,28,30–34]. It was generally agreed that LPMOs utilise either an O_2_-derived Cu(II)-superoxide or a Cu(II)-oxyl intermediate to oxidise their substrates, with the generation of the latter requiring multiple electrons in order to cleave the O-O bond (see reviews [22,35,36]). Recent research, however, has called into question the physiological relevance of these mechanisms by proposing that H_2_O_2_ (the redox-state equivalent of O_2_ + 2H^+^ + 2e^−^) may be the true co-substrate for LPMOs [23,37–39]. Such proposals were originally based on an observed increased rate of LPMO reaction when using H_2_O_2_ for activity as opposed to O_2_ [23,37–39]. Further support for this proposal has also come from computational studies which suggest that using H_2_O_2_ as a co-substrate is more energetically favourable than using O_2_ [40–43]. Whether H_2_O_2_ is the true co-substrate, or a ‘reaction-shunt’ (i.e. H_2_O_2_ provides a shortcut to forming the same key reactive intermediate required in an O_2_ and electron dependent reaction) is difficult to dissect, not least because hydrogen peroxide is known to cause significant oxidative damage to LPMOs [23,37,44,45], and also because lab-based studies can be misleading in terms of the multiple components that might be involved in activating LPMOs [46].

Whether O_2_ or H_2_O_2_ is the co-substrate, LPMOs require a source of electrons for activity in order to induce the reduction of the active site Cu^II^ to Cu^I^. In laboratory experiments, small molecule reducing agents are typically used to do this and trigger LPMO action. In fungal LPMO systems, several enzymes including cellobiose dehydrogenase (CDH) [47–50], glucose-methanol-choline (GMC) oxidoreductases [50,51] and AA12 pyrroloquinoline quinone (PQQ)-dependent pyranose dehydrogenases [52], have also been demonstrated to donate electrons to LPMOs thereby activating them for action. Since CDH and AA12 enzymes have an appended *b*-type cytochrome domain (AA8) which mediates the electron transfer to the LPMO, a direct interaction between enzymes must occur [52,53]. *In silico* docking [52,53] and protein–protein interaction studies [54] so far suggest that these enzyme partners directly contact the copper ion to allow its reduction, but the transient nature of the interaction and the difficulty in assessing these interactions in the presence of the substrate, has made it challenging to characterise this further. Indeed, several studies have proposed the presence of electron hopping pathways through the core of the LPMO [6,30,32,55,56], which may be of particular relevance in cases where an LPMO activating enzyme partner has been invoked as the electron source. There are many open questions that remain to be addressed in this area, but what is clear is that the activity of LPMOs is only fully revealed when they act as part of a consortium of enzymes, not in isolation. Determining how the individual components of the consortium interact and what their roles are is perhaps the most important question facing the LPMO field, and whether such systems also operate outside of the fungal kingdom is a key outstanding question to be addressed.

While a native bacterial LPMO protein partner has yet to be identified, two bacterial proteins have been suggested as candidates, these are Cbp2D and Cbp2E from *Cellvibrio japonicus* [57]. When Gardner and co-workers knocked out the genes coding for these proteins, the growth of the resulting *C. japonicus* strain was significantly retarded on cellulose filter paper, suggesting they played a prominent role in cellulose metabolism. Additionally, both proteins have been detected in secretomic studies during growth on chitin, suggesting a potential role in the general polysaccharide metabolism of this organism [58]. Both Cbp2D and E contain a carbohydrate-binding module (CBM2) and a YceI-like ubiquinone-8 binding domain, designated an X158 domain, as described by Vincent *et al.* [57,59]. Ubiquinone-8 is well known for its role in the electron transport chain hinting that proteins harbouring this domain may have a redox function [59]. Additionally, Cbp2D also harbours two predicted *c*-type cytochrome domains at its C-terminus suggesting an electron transfer function for this protein, but direct links between these proteins to LPMO biochemistry have not yet been demonstrated experimentally [57].

We therefore set out to characterise Cbp2D structurally and biochemically to better understand its likely function. Here, we present the structure of a domain from Cbp2D, which we dub *Cj*X183, confirming that it is indeed a small *c*-type cytochrome domain, with an exposed heme cofactor. We further show that the isolated domain is redox active and can activate two bacterial LPMOs for oxidative action on cellulose. By comparing the results of LPMO activity using *Cj*X183 as a reductant to those using ascorbate, we highlight differences that may relate to how a small molecule may act compared with a protein-based electron source. Our results therefore highlight the need for a better understanding of the roles of redox partners to LPMOs which may help unravel further details of how these enzymes are harnessed in nature.

## Results

### Bioinformatic analysis of Cbp2D reveals several domains of unknown function

**Figure 1.**
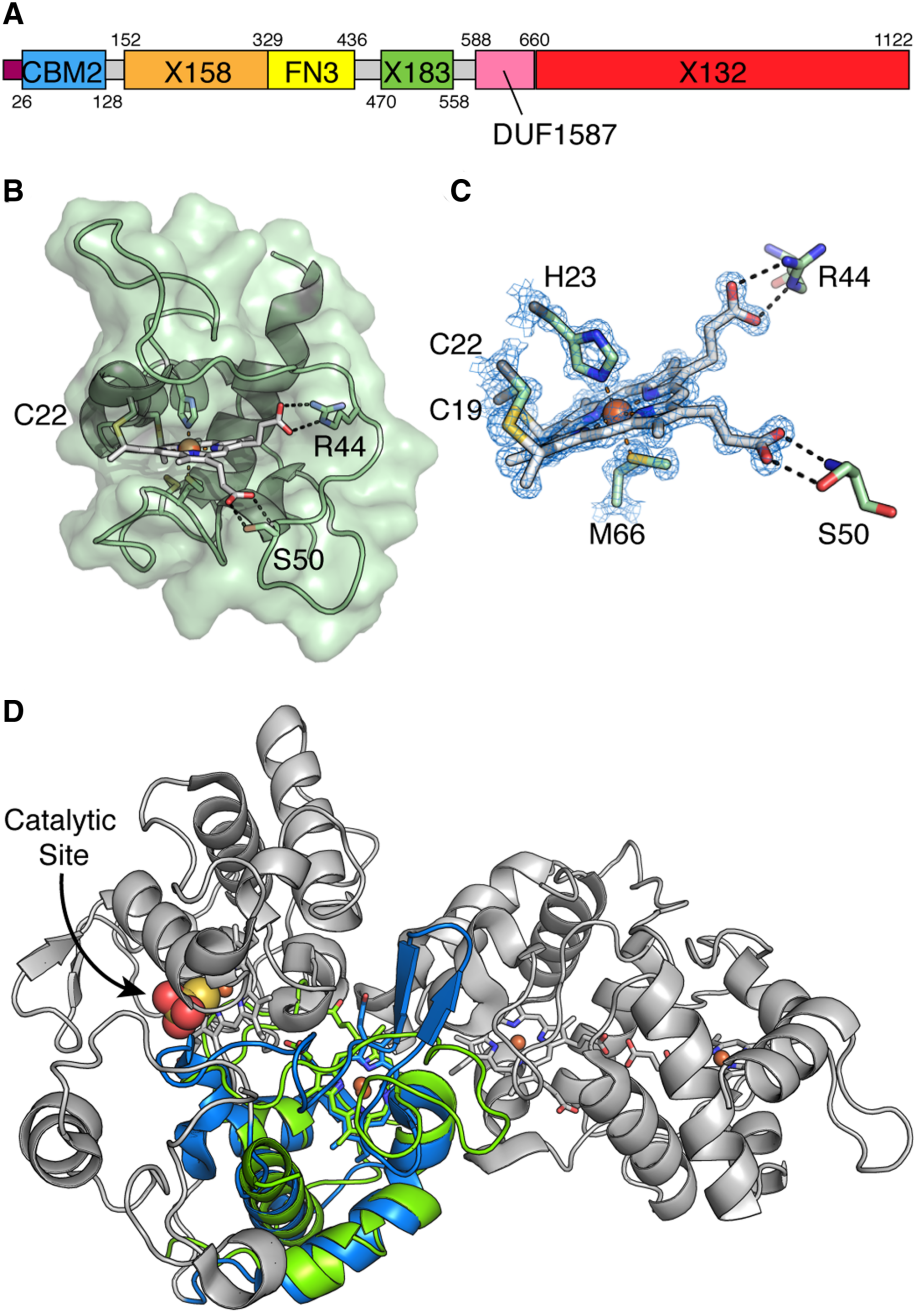
Structural analysis of *Cj*X183. (**A**) Domain annotation for Cbp2D used throughout this work. Numbers indicate the point in the linear sequence at which the domain boundaries reside. X domains represent domains of unknown function whilst FN3 and DUF1587 domains are annotated in pfam (see Supplementary Spreadsheet S1 for further examples of proteins that harbour these domains). (**B**) Overall structure of *Cj*X183 displayed as a cartoon with the protein surface shown in green. The heme molecule bound to the protein is shown as sticks with white carbon atoms and the central iron atom shown as an orange sphere. Important residues that interact with the heme molecule are also shown as sticks coloured by atom type with green carbon atoms. (**C**) Close up view of the heme molecule in *Cj*X183. The 2F_o_–F_c_ map is shown as a blue mesh around the heme molecule and coordinating residues contoured at 1σ. (**D**) Superposition of *Cj*X183 (green) with TsdBA from *Marichromatium purpuratum* (PDB ref 4v2k, [60]). The domain in TsdBA with which *Cj*X183 superposes is coloured blue with the rest of the protein coloured grey. The catalytic site in TsdBA is indicated by the molecule of thiosulfate shown as spheres. The chain of heme molecules in TsdBA, shown with grey carbons, are proposed to shuttle electrons away from the active site following catalysis [60].

Gardner *et al*. have shown previously that *cbp2D* codes for a protein that is predicted to contain a carbohydrate-binding module, a YceI-like quinone binding domain, a fibronectin type III (FN3) domain and two *c*-type cytochrome domains [57]. To better define the domain boundaries for these, we performed a sequence analysis of Cbp2D to generate the annotation shown in Figure 1A. Vincent *et al.* defined the YceI-like domain in a protein similar to Cbp2E from *Saccharophagus degradans* as an X158 domain. This domain has been demonstrated to bind to polyisoprenoids, with one of the two structures determined containing ubiquinone-8 (UQ-8), suggesting a potential redox role for proteins containing these domains [59]. Likewise, Gardner *et al.* [57] determined that Cbp2D contains two domains at the C-terminus which encompass distinct sequences of different lengths, but both harbour a single CXXCH motif indicative that they are *c*-type cytochrome-like domains. We have defined these as X183 and X132 domains, in line with the naming convention for X158, as these represent domains of unknown function but are regularly associated with CBMs and other domains that are known to CAZy (for further examples of proteins harbouring these domains see the Supplementary Spreadsheet S1) [17]. Given the apparent redox nature of these proteins, we set out to better structurally and biochemically characterise Cbp2D from *Cellvibrio japonicus* with the aim of determining this protein's potential to act as an LPMO electron donor. We were unable to produce the full-length protein or express a range of constructs coding for the individual X-domains encoded within the linear sequence. The X183 construct, however, produced protein at high-levels and herein we describe our characterisation of this domain.

### *Cj*X183 is a small *c*-type cytochrome-like domain

*Cj*X183 was expressed in the periplasm of *E. coli* with the aid of the pEC86 vector which codes for the cytochrome maturation machinery to allow the insertion of heme into the protein [61]. The red coloured protein was subsequently purified to homogeneity, crystallized and the structure determined to 1.2 Å resolution (see Supplementary Table S1 for structure solution and refinement statistics). The structure reveals *Cj*X183 to be an α-helical *c*-type cytochrome domain (Figure 1B) harbouring a single heme molecule, which is covalently linked to the conserved CXXCH motif via thioether linkages to Cys19 and Cys22. Two additional cysteines (residues 74 and 78) are present towards the C-terminus of the domain and these form a disulfide bond, presumably to stabilise the protein in the extracellular environment. The central heme iron atom is coordinated by a proximal histidine residue, His23, and a distal methionine residue, Met66 (Figure 1C) in the typical configuration observed for class one *c*-type cytochromes [62]. Interestingly, the edges of the heme molecule appear to be largely solvent exposed (Figure 1), however, the propionate groups form hydrogen bonds with Arg44 and Ser50 located in a surface loop which extends around this part of the molecule. These interactions may account for the relative stability of the reduced state discussed later.

Structural comparisons against the PDB were performed using the DALI server [63]. *Cj*X183 aligns with a number of other small *c*-type cytochrome domain-containing proteins with the closest match being a single domain from the thiosulfate dehydrogenase/tetrathionate reductase, TsdBA, from *Marichromatium purpuratum* (PDB ref 4v2k, [60]). The superposition reveals an rmsd of 2.3 Å between the proteins over 72 Cα positions and a sequence identity of only 31% (Figure 1D). TsdBA in this organism is an unusual gene fusion in which the genes that typically code for the proteins TsdA and TsdB have been fused, coding for their expression in a single polypeptide chain. A series of *c*-type cytochrome domains in these proteins form an electron relay, which feeds electrons generated by the oxidation of thiosulfate into either photosynthetic or respiratory electron transfer chains [60]. Within TsdA, there are two *c*-type cytochrome domains, one of which has unusual coordination of the heme iron via a cysteine and histidine and is the catalytic site at which thiosulfate is converted to tetrathionate. The second domain is responsible for shuttling electrons away following catalysis to be utilised elsewhere in respiration/photosynthesis. *Cj*X183 aligns to the electron transfer domain within TsdA, suggesting a similar electron transfer role for *Cj*X183 as is typical of this cytochrome class (Figure 1D). It should be noted that in TsdBA the heme propionate groups of the domain that *Cj*X183 superposes with are ideally positioned for receiving electrons from the TsdA active site heme. It is therefore possible that *Cj*X183 may be similarly positioned with reference to the other domains within the full-length Cbp2D, playing a role in shuttling electrons through the protein. Without a structure of the full-length protein, we cannot determine whether this is the case, and so we decided to investigate the redox properties of the domain in isolation as a first step to determining whether Cbp2D may have a role in LPMO activation.

### *Cj*X183 can donate electrons to LPMOs and activate them for cellulose degradation

**Figure 2.**
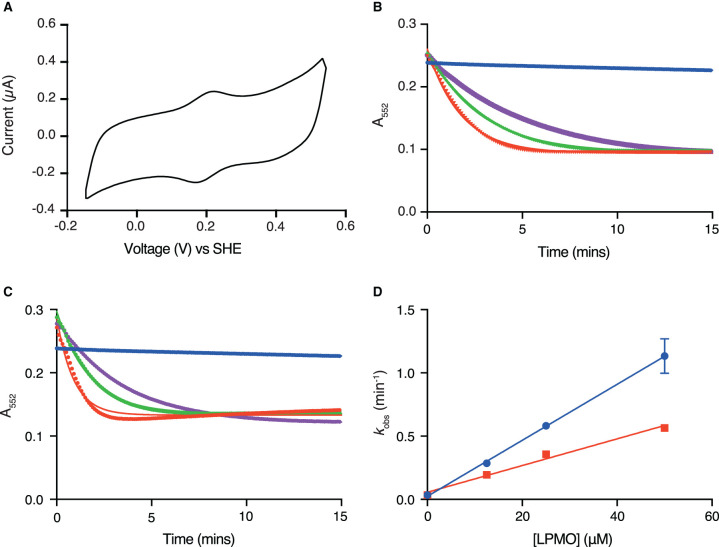
Analysis of *Cj*X183 redox reactivity. (**A**) Cyclic voltammetry measured for *Cj*X183 on a graphite electrode reveals a reduction potential of +193 mV vs SHE. (**B**) Monitoring of the reduced state in *Cj*X183 as measured by the A_552_ over time in the absence of *Cj*AA10BΔCBM (blue), and then with 1.25 fold (purple), 2.5 fold (green) and 5 fold (red) excess of *Cj*AA10BΔCBM. (**C**) Equivalent plot as panel B for *Cf*AA10. (**D**) Plot of oxidation rates for *Cj*X183 against *Cj*AA10BΔCBM (red) or *Cf*AA10 (blue) concentration show a linear relationship.

Protein film voltammetry was used to assess the likelihood that *Cj*X183 could activate LPMOs. As shown in Figure 2A, when *Cj*X183 is adsorbed onto a graphite electrode and the voltage is swept between −0.15 and +0.54 V vs SHE at pH 7.0 and 5°C, clear peaks centred around ∼0.2 V vs SHE are observed for the oxidation (positive current) and reduction (negative current) of the protein domain. Analysis of these signals (Supplementary Figure S1 and Supplementary Notes [64,65]) indicates that they arise from a one-electron redox reaction that is attributed to the reversible Fe^III/II^ chemistry of the heme within *Cj*X183. This experiment suggested that in principle *Cj*X183 is capable of activating LPMOs because the extracted midpoint potential (+193 mV vs SHE, Supplementary Table S2) sits below the reduction potentials that have been reported for LPMOs (typically greater than +220 mV vs SHE)[28,31,66–68]. We sought to investigate this further biochemically.

*Cj*X183 demonstrates a typical UV-vis absorption spectrum for a *c*-type cytochrome with an intense Soret band at 413 nm in the oxidised state, which shifts to 419 nm upon reduction, concomitant with the appearance of the α and β bands which have absorption maxima at 523 nm and 552 nm, respectively (Supplementary Figure S2). These spectroscopic properties make it easy to monitor the redox state of *Cj*X183 by measuring the absorbance at 552 nm (A_552_)[62]. *Cj*X183 was reduced using ascorbate and then passed down a PD-10 desalting column to remove any excess reducing agent. The UV-Vis spectrum was then monitored over time whilst the sample was exposed to air. The complete oxidation of the heme in *Cj*X183 took ∼2 h demonstrating that it remains reduced in the presence of O_2_ for an extended period, even though the structure reveals that the heme is quite solvent exposed. This is likely the result of the heme propionate groups showing close interactions with a loop that may help stabilise the reduced state.

Since *Cj*X183 appeared to maintain its reduced state in an aerobic environment, we used the UV-Vis spectrum to investigate the effect of the addition of LPMOs on the rate of heme oxidation. For these and subsequent experiments, two LPMOs were used. The cellulose active LPMO from *Cellvibrio japonicus*, *Cj*AA10B, which has been previously investigated by Gardner *et al.* [57], and an LPMO from *Cellulomonas fimi*, *Cf*AA10, characterised by Crouch *et al.* [69]. Both enzymes typically possess C-terminal carbohydrate binding modules but in order to produce enough enzyme for our work we expressed a truncated version of the *C. japonicus* enzyme lacking its CBM which will be referred to as *Cj*AA10BΔCBM henceforth. Upon addition of each LPMO, *Cj*X183 demonstrated an increased rate of oxidation as demonstrated by a more rapid decrease in A_552_ (Figure 2B,C). Fitting these data to a single exponential provided the rate of decay of the reduced state. The rate of *Cj*X183 oxidation by oxygen is close to zero over this timescale so was assumed not to affect the assay. As the concentration of *Cj*AA10BΔCBM or *Cf*AA10 was increased from 0 to 50 µM, the oxidation rate of *Cj*X183 also increased linearly up to a highest rate of 0.56 min^−1^ for *Cj*AA10BΔCBM and 1.13 min^−1^ for *Cf*AA10 (Figure 2D). These rates are considerably slower than those reported by Loose *et al.* [70] for electron transfer between MtCDH and a bacterial AA10 (observed electron transfer rate of 32 s^−1^ when a 1 : 10 molar ratio of CDH:AA10 was used), which may suggest a different mode of interaction between the proteins here. The results of this assay, nonetheless, suggest an enzyme-dependent electron transfer event from reduced *Cj*X183 to both of the LPMOs tested. The rate of oxidation appeared to be faster when using *Cf*AA10 as opposed to the enzyme from the same species as *Cj*X183. The reason for this is currently unclear, but these results appeared to further support the notion that *Cj*X183 could activate these enzymes (and others) for cellulose degradation.

**Figure 3.**
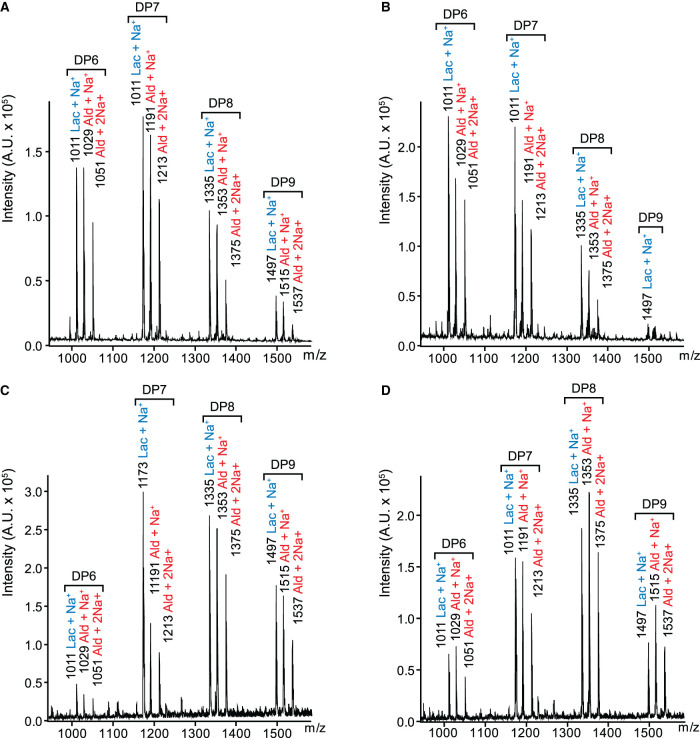
MALDI-ToF MS analysis of *Cf*AA10 and *Cj*AA10ΔCBM enzymatic products. C1 oxidised oligosaccharide products released from PASC when (**A**) using ascorbate to activate *Cj*AA10BΔCBM; (**B**) using ascorbate to activate *Cf*AA10; (**C**) using *Cj*X183 to activate *Cj*AA10BΔCBM; and (**D**) using *Cj*X183 to activate *Cf*AA10. In all panels the m/z of each peak is annotated together with the oxidised product formed — Lac representing oligosaccharides with a reducing end lactone, and Ald representing oligosaccharides with an aldonic acid at their reducing ends. DPn (where *n* is an integer) indicates the length of the oligosaccharide products.

Activity assays with both LPMOs were carried out on phosphoric acid swollen cellulose (PASC) in which either ascorbate or reduced *Cj*X183 were used as the electron source. Following overnight incubation of the reaction components, samples were analysed by MALDI-TOF mass spectrometry to detect the presence of oxidised oligosaccharide products arising from LPMO activity (Figure 3). Controls using ascorbate as the reducing agent clearly liberated typical lactone and aldonic acid terminated oligosaccharides (Figure 3A,B), as had been observed previously with these LPMOs [57,69]. Importantly, when chemically reduced *Cj*X183 was used as the electron donor a similar product profile was observed with both LPMOs (Figure 3C,D). To check that the observed activity was due to electron donation from *Cj*X183, and not any residual ascorbate that may remain following desalting, we also used electrochemically reduced *Cj*X183, which had not been exposed to chemical reducing agents, to test the activity of *Cf*AA10 (Supplementary Figure S3). The results of this experiment confirmed the same result, giving a clear demonstration that reduced *Cj*X183 alone can induce LPMO activity on cellulose.

### Use of *Cj*X183 as an electron source results in less hydrogen peroxide production by the LPMO

It is now well established that LPMOs will turnover oxygen to hydrogen peroxide in the presence of chemical reducing agents and the absence of a polysaccharide substrate [71,72]. This ‘side’ activity has been used as a proxy for measuring LPMO activity where direct kinetic measurements of cellulose degradation has not been possible, so we were interested to see how our X183 domain compared with small molecule reducing agents in such conditions. We therefore used the well-established Amplex Red H_2_O_2_ assay [71–73] to detect H_2_O_2_ production by both *Cj*AA10BΔCBM and *Cf*AA10 in the presence of ascorbate or reduced *Cj*X183. We took great care to perform controls using the reducing agents in the absence of the LPMOs and to take into account any absorbance effects resulting from the presence of the *Cj*X183 in the preparation of H_2_O_2_ standard curves to quantitate the assay. Since the reductants were not being regenerated during these experiments, *Cj*X183 was used as a one electron donor and ascorbate as a two-electron donor, consequently, *Cj*X183 was used at twice the concentration of ascorbate to ensure the same number of electrons were available to the LPMO in each reaction.

**Figure 4.**
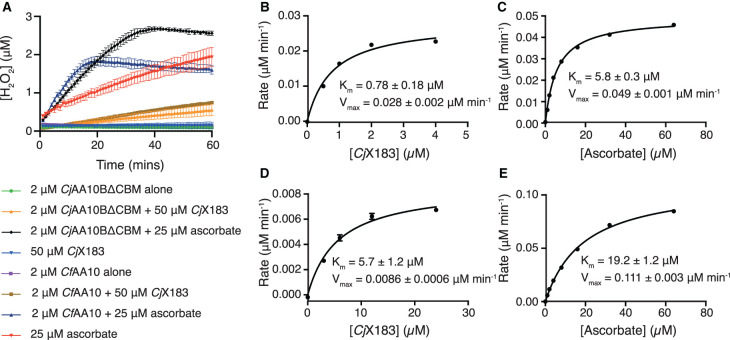
Analysis of H_2_O_2_ production using the Amplex Red H_2_O_2_ assay. (**A**) plot of the H_2_O_2_ concentration against time for samples as indicated by the inset. (**B**) Michaelis–Menten plot for H_2_O_2_ production by *Cj*AA10BΔCBM when activated by *Cj*X183, (**C**) *Cj*AA10BΔCBM when activated with ascorbate, (**D**) *Cf*AA10 activated with *Cj*X183 (**E**) *Cf*AA10 activated by ascorbate.

In the absence of either LPMO, the natural autooxidation of ascorbate produced significantly more H_2_O_2_ compared with *Cj*X183 alone (Figure 4A). This is in line with other's findings which highlight that ascorbate produces significant levels of H_2_O_2_ which may be utilised by the LPMO in its reaction [23,46,72]. Our controls lacking LPMO, also further highlight *Cj*X183's ability to remain in its reduced state following removal of exogenous reducing agents. A marked increase in H_2_O_2_ production stemming from LPMO activity was observed in the presence of *Cj*AA10BΔCBM and *Cf*AA10 using both reducing agents (Figure 4A). For both *Cj*AA10BΔCBM and *Cf*AA10, activation using reduced *Cj*X183 produced significantly less H_2_O_2_ after an hour of incubation compared with the use of ascorbate — 0.55 µM and 0.75 µM for *Cj*AA10BΔCBM and *Cf*AA10, respectively, compared with 2.56 µM and 1.6 µM when using ascorbate (Figure 4A).

To further dissect the differences in H_2_O_2_ production by these enzymes we attempted to perform a more detailed kinetic analysis using this assay. *Cj*AA10BΔCBM and *Cf*AA10 concentrations were held constant while the concentration of *Cj*X183, or ascorbate, was varied around calculated *K*_M_ values, with H_2_O_2_ production measured over time. Initial rates from the first 10 min were extracted and plotted against reductant concentration, giving the data shown in Figure 4B–E. Under these conditions, the rate of H_2_O_2_ production by both enzymes appeared to obey Michaelis–Menten kinetics and the data fit well to such an analysis (Figure 4B–E and Supplementary Table S3). As was expected from previous experiments, the maximal rate of H_2_O_2_ production by both LPMOs was achieved when ascorbate was used as the electron source. This was 2- and 10- fold lower when reduced *Cj*X183 was used for *Cj*AA10BΔCBM and *Cf*AA10, respectively. Comparing the *K*_M_ values that were extracted for each LPMO reductant pairing also shows that the *K*_M_ is consistently lower when *Cj*X183 is used as the reductant compared with ascorbate. Whilst this is a complicated reaction and there may be many explanations for the observed kinetic constants, if one assumes that the *K*_M_ reports on the affinity between the reductant and LPMO, these data may hint that there is a higher affinity, and potentially longer-lived interaction between *Cj*X183 and *Cj*AA10BΔCBM, which may account for the differences in maximal H_2_O_2_ production rates observed for this pairing as compared with ascorbate. Furthermore, the maximal rate of H_2_O_2_ production is ∼3-fold higher in the *Cj*X183–*Cj*AA10ΔCBM pairing compared with when *Cj*X183 is used to activate *Cj*AA10. It is, therefore, tempting to speculate that these proteins may have evolved to interact with one another, but this will require further verification using other techniques. Whether this turns out to be true or not, there were clearly differences in the performance of the LPMO in producing H_2_O_2_ when comparing the use of the *Cj*X183 *c*-type cytochrome to ascorbate, which both represent similarly finite electron sources which were not being replenished.

### Using *Cj*X183 as reductant results in less protein damage

**Figure 5.**
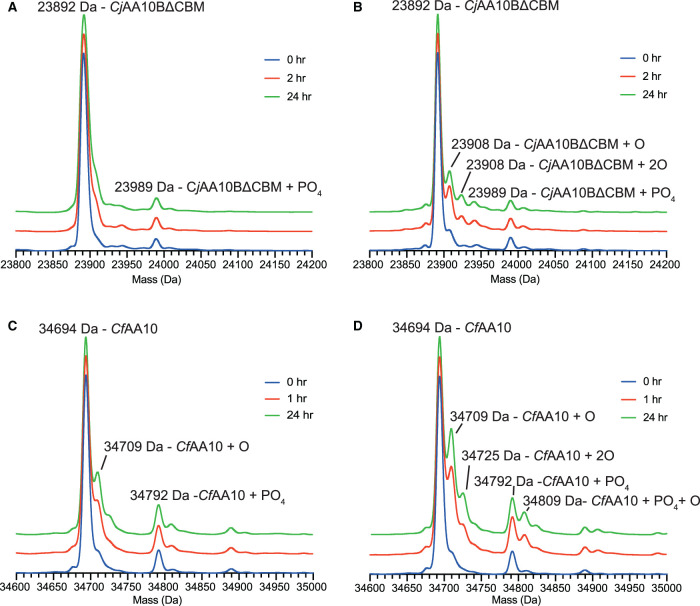
Protein damage analysis by electrospray (ESI) mass spectrometry. (**A**) ESI mass spectra for *Cj*AA10BΔCBM at the indicated time points following incubation with *Cj*X183. (**B**) ESI mass spectra as taken for panel A in which the electron source was replaced with an equivalent quantity of ascorbate. (**C**) ESI mass spectra for *Cf*AA10 at the indicated time points after incubation with *Cj*X183. (**D**) ESI mass spectra as taken for panel C in which the electron source was replaced with an equivalent quantity of ascorbate.

Recent studies have suggested that there is a delicate balance to be struck between the concentrations of H_2_O_2_ and reductant to ensure that the LPMO is maximally active for as long as possible [23,37–39,74]. Given the apparent lower level of H_2_O_2_ production that we observed when using *Cj*X183 as the electron source, we reasoned that this would likely result in less damage to the LPMOs themselves. We therefore used electrospray ionisation mass-spectrometry (ESI-MS) to assess this by incubating the enzymes with reduced *Cj*X183 or ascorbate as would be done during an activity assay, but in the absence of cellulose. Accurate ESI-MS spectra were then taken for the proteins at set time points to assess whether the protein was accumulating oxidative damage over time. The resulting spectra revealed the appearance of +16 Da species in both the *Cj*AA10BΔCBM and *Cf*AA10 spectra, which build in over time, indicative of direct oxidation of the enzyme (Figure 5). Using the height of the oxidised protein peaks relative to the native protein peak as an indication of the level of oxidative damage to the sample, it appears that the *Cf*AA10 enzyme is much more prone to damage than the *Cj*AA10BΔCBM protein (cf Figure 5A,B with 5C,D). Both enzymes also show fewer signs of damage when *Cj*X183 was used as the reductant compared with ascorbate (cf Figure 5A,C with 5B,D), with *Cj*AA10BΔCBM appearing almost unaltered after 24 h when activated by the reduced *Cj*X183 domain (Figure 5A). These results correlate well with those from the Amplex Red H_2_O_2_ assays in which *Cf*AA10 was found to produce the most H_2_O_2_ under our assay conditions and had the highest *V*_max_ when ascorbate was used as the reductant, establishing a direct link between the production of H_2_O_2_ and oxidative damage to the protein in line with other studies [23,37–39,74].

### Effective glycoside hydrolase boosting is maintained using *Cj*X183 as the electron source

**Figure 6.**
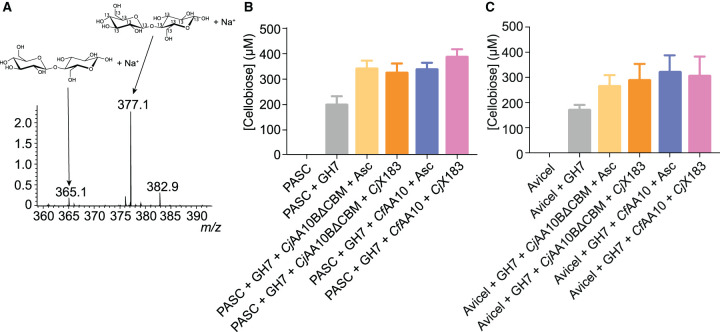
Activity boosting of LPMOs on cellulose. (**A**) Example spectrum for quantification of cellobiose (365 m/z species) by reference to a known quantity of ^13^C-labelled cellobiose (377.1 m/z species). (**B**) Histogram showing the quantity of cellobiose released by *T. longibrachiatum* GH7 in the absence and presence of *Cj*AA10BΔCBM and *Cf*AA10 with differing electron source on PASC and (**C**) on Avicel.

Our analysis of the effect of using reduced *Cj*X183 compared with ascorbate on the production of H_2_O_2_ by LPMOs suggested that the protein partner might be a less efficient LPMO activating agent, but that this may result in less oxidative damage to the enzyme. We therefore sought to perform boosting experiments with a cellulase to assess whether using *Cj*X183 as the source of electrons to the LPMO had an overall influence on the efficiency with which cellulose can be degraded in an enzyme cocktail. Cellulose degradation assays were therefore performed on PASC and Avicel using a GH7 cellobiohydrolase from *Trichoderma longibrachiatum* in the presence of *Cj*AA10BΔCBM or *Cf*AA10 and either ascorbate or reduced *Cj*X183 as the electron source. The cellobiose that was released from the substrate was then quantified by adding a known amount of ^13^C-labelled cellobiose to each sample and analysing the reaction products using ESI-MS (Figure 6A). Under the conditions we tested, we observed an ∼2-fold increase in cellobiose release in the presence of both *Cj*AA10BΔCBM and *Cf*AA10 on both substrates used (Figure 6B,C), as has been demonstrated many times for LPMOs [4–9]. Furthermore, a similar amount of cellobiose was produced by the GH7 in the presence of each LPMO irrespective of the electron source (Figure 6B,C), and hence the level of activity boosting remained the same. These results suggest that even though the *Cj*X183-driven activation of LPMO appears slower and results in less H_2_O_2_ production, the overall effectiveness of the LPMO as a cellulase booster is maintained under these conditions when *Cj*X183 is used as the reductant. This may therefore indicate that the LPMO remains active for longer when *Cj*X183 is used as the activator resulting in a similar level of boosting over the period of the experiment.

## Discussion

Much of the focus on LPMO biochemistry has recently shifted towards a model in which H_2_O_2_ is the active co-substrate used by these enzymes during the oxidative deconstruction of cellulose [23,38,39]. The rate at which LPMOs act is significantly improved through H_2_O_2_ supply, but importantly, the H_2_O_2_ concentration needs to be carefully controlled to ensure that the enzyme is not inactivated by oxidative damage [37]. The proclivity of the reductant to generate H_2_O_2_ is another topic that has come into consideration, with studies demonstrating that ascorbate particularly generates significant levels of H_2_O_2_ which can be harnessed by the LPMO for activity [23,46,72]. Protein partners have also been widely considered as electron sources for LPMOs, with CDH representing the key player considered in this context. CDH can generate H_2_O_2_, but also harbours a *b*-type cytochrome domain which allows it to directly transfer electrons to LPMOs [47–50,75]. Bacteria are not known to possess an enzyme equivalent to CDH and candidate enzymes that may play a similar role have not been widely investigated representing a key gap in our knowledge of how LPMOs are optimally harnessed in these microbes. Cbp2D and Cbp2E are two proteins that have been suggested as candidates to play this role in bacteria, but biochemical confirmation of this has not yet been possible [57]. We therefore set out to better characterise the potential function for a portion of one of these multi-modular proteins through structural and biochemical analysis.

Cbp2D contains at least three domains (X158, X183 and X132) that appear to have a likely redox function. Here, we have determined the structure of the isolated X183 domain (*Cj*X183) from this protein, confirming it as a typical *c*-type cytochrome that most likely has an electron transferring (rather than a catalytic) function. Whilst we were unable to study *Cj*X183 as part of the larger Cbp2D protein, we have demonstrated that *Cj*X183 alone can act as an electron donor to two bacterial LPMOs, supporting their cellulose oxidising activity. The apparent rates of electron transfer between *Cj*X183 and the two LPMOs studied here were rather slow (Figure 2) and are considerably slower than those observed when *Mt*CDH was used to activate an AA10 from *Streptomyces coelicolor* [70]. The observed differences in rates could be driven by the different reduction potentials of these proteins/domains. We measured the reduction potential for *Cj*X183 to be +193 mV vs SHE, which is significantly higher than those reported for the haem in CDH which are typically between +90 and +150 mV vs SHE [47]. Given that LPMOs typically have reduction potentials above +220 mV vs SHE, the driving force for electron transfer between CDH and an LPMO will be greater compared with that for *Cj*X183 which may account for this. In addition, there may be differences in the mode of interaction between the proteins which will influence the electron transfer rate. Where studied, the interaction between CDH and LPMOs has been proposed to occur by direct contact between the haem and the copper ion in the LPMO active site [53,54]. It is unknown whether *Cj*X183 is likely to approach the LPMO in the same manner, and the surface properties of the protein may also influence the mode of interaction which could also affect the electron transfer rate. These data do not discount a potential role for this domain, or Cbp2D more broadly, as an electron donor to LPMOs and so we were interested to investigate how this apparent slow electron donation from the X183 may influence LPMO activity.

Since *Cj*X183 was not linked to an enzymatic activity, we could directly compare this domain's ability to activate LPMOs with that of ascorbate, which would represent a similarly finite electron source, not replenished during LPMO activity assays. We reasoned that this may report on differences relating to the use of a protein-based electron donor compared with a small molecule reductant. We used the ability of the LPMO to produce H_2_O_2_ as a means of assessing the efficiency with which electrons were delivered to the enzyme from the different reductants. The kinetic data from this analysis are complex and need careful interpretation given the range of potential rate-limiting steps under consideration. These include the rate of O_2_ binding to the LPMO, the rate of electron transfer between electron source and LPMO, as well as the affinity and lifetime of a productive interaction between the electron source and LPMO. However, there are clear differences in the rate of H_2_O_2_ production by each LPMO in response to the different electron sources studied. Of note, when *Cj*X183 was used as the electron source the *K*_M_ values extracted from the kinetic analysis were lower compared with those revealed for ascorbate. If one considers that these *K*_M_s may report on the affinity of the interaction between the electron source and LPMO, it may be that there is a higher affinity interaction between the proteins compared with the interaction between ascorbate and the LPMO. If this is the case, then the highest affinity interaction seems to occur between *Cj*X183 and the LPMO from the same species: *Cj*AA10BΔCBM. Taken together with the findings discussed above, these data suggest that there may be a specific interaction between these proteins, which we are currently investigating in greater detail. If we can confirm that a specific interaction exists between these proteins, then this may lend further support to the notion that Cbp2D's role is to activate LPMOs for action.

Having established that *Cj*X183 can be used to activate two LPMOs to catalyse cellulose oxidation, we were interested in the potential consequences of *Cj*X183's apparent diminished ability to induce H_2_O_2_ production by the LPMO relative to ascorbate. Examining protein damage, we found that the reductant-LPMO pairing that resulted in least H_2_O_2_ production also resulted in the least oxidative damage to the LPMO. This is also in line with H_2_O_2_ dosing studies which have clearly established that this species directly damages the LPMO when used in reactions [23,37–39]. During our analyses we took care to ensure that the same number of electrons were available to the LPMO, irrespective of whether ascorbate or reduced *Cj*X183 was used as the electron source, so it is not simply the number of electrons available that determine the H_2_O_2_ production level by the LPMO. As might be expected, H_2_O_2_ production must be influenced by other factors including the reduction potentials of the species involved and the mode of interaction between the LPMO and reductant. The use of a protein partner as an electron source may, therefore, have significant effects upon the LPMO reaction and so attention should continue to be paid to the role of protein partners in LPMO biochemistry.

Irrespective of whether ascorbate or *Cj*X183 was used as the activator, there was no significant difference in the amount of cellobiose liberated from either PASC or Avicel by a GH7 cellobiohydrolase in the presence of the LPMOs used during this study. This is somewhat reflective of studies where boosting has been examined in the context of the effect of H_2_O_2_ feeding on overall saccharification. Whilst LPMO activity has been shown to be significantly improved in terms of kinetics when H_2_O_2_ is provided as the co-substrate [23,38,39], only modest improvements in boosting were observed by Müller *et al.* [37] in carefully controlled H_2_O_2_ feeding experiments. This likely reflects the fact that it is the cellulase kinetics that ultimately define the rate at which cellulose is degraded overall. The LPMO reaction clearly benefits these enzymes but there may be a limit to those benefits, and there is a likely balance to be struck between the level of substrate oxidation and protein damage occurring during LPMO use.

In conclusion, we have demonstrated that a domain from Cbp2D in *C. japonicus* is capable of activating LPMOs for cellulose deconstruction and have used this to examine differences in LPMO activity resulting from the use of a finite electron source from either a protein or small molecule. We have been able to demonstrate that the choice of electron source can influence H_2_O_2_ production by the LPMO, which was directly correlated to protein damage under the conditions that we tested. Whether Cbp2D's physiological function is to activate LPMOs remains an open question that we are currently examining, and how such an activity could be coupled to an electron generating reaction remains a key question. What is clear, is that Cbp2D must have some electron transfer capacity and based on other studies has a key role during cellulose deconstruction [57], so further study of this and related proteins should continue to be a high priority for the field.

## Experimental procedures

### Expression and purification of *Cj*X183

*Cj*X183 (bases 1408 to 1674 from the *cbp2d* gene in Genbank CP000934.1) was sub-cloned from a previously generated pET-26b construct which incorporated a pelB leader sequence for periplasmic protein export and C-terminal His_6_ tag into the pCW-LIC vector (a gift from Cheryl Arrowsmith, Addgene plasmid 26098) using PIPE cloning [76]. The pCW-*Cj*X183 plasmid was used to transform *E. coli* BL21(DE3) cells already transformed with the pEC86 plasmid, encoding the heme maturation system [61]. 1 L cultures of 2TY media (16 g/L tryptone, 10 g/L yeast extract, 6 g/L NaCl) were grown to an A_600_ of 0.6 at 37°C shaking at 200 rpm, cooled to 20°C and induced with a final concentration of 0.4 mM IPTG. Cultures were grown for 20 h before harvesting by centrifugation at 5000×***g*** for 20 min at 4°C.

Cells were resuspended in three volumes of ice-cold lysis buffer (50 mM Tris pH 8, 200 mM NaCl, 20% w/v sucrose) to which 40 µl of hen egg white lysozyme was added per gram of cell paste and incubated on ice for 1 h. 60 µl of 1 M MgSO_4_ was then added per gram of cell paste and the lysate was incubated for a further 20 min on ice. The suspension was centrifuged at 10 000×***g*** for 20 min at 4°C and supernatant containing the periplasmic fraction was removed. The pellet was then resuspended in three volumes of ice-cold water, left on ice for 1 h and centrifuged as above. The supernatants from the two lysis steps were pooled and protein purified from here on a 5 ml HisTrap FF (GE Healthcare) column equilibrated with buffer A (50 mM Tris pH 8, 200 mM NaCl, 30 mM imidazole). Protein was eluted from the column using a linear gradient of 0 to 100% buffer B (50 mM Tris pH 8, 200 mM NaCl, 300 mM imidazole) over 10 column volumes collecting 1.6 ml fractions. Peak fractions containing *Cj*X183 were pooled and concentrated to 1 ml using a Vivaspin 3000 Da cut off concentrator at 4000×***g***. Concentrated protein was applied to a HiLoad 16/600 Superdex S75 column (GE Healthcare) equilibrated in GF buffer (50 mM Tris pH 8, 200 mM NaCl) collecting 1.6 ml fractions after the void volume had been eluted. Peak fractions containing the purified *Cj*X183 were combined, concentrated on the same concentrator, and the sample was then quantified using the A_410_ extinction coefficient for heme *c* of 106 000 M^−1^ cm^−1^.

### Expression and purification of *Cj*AA10BΔCBM and *Cf*AA10

The pET-22-*Cj*AA10BΔCBM construct was generated by deletion of the nucleotide sequence coding for the CBM region of the pET22-*Cj*AA10B construct provided by Gardner *et al.* [57] using the Q5 site-directed mutagenesis kit (New England Biolabs). Following this, *Cj*AA10BΔCBM and *Cf*AA10 were expressed as described by Gardner *et al.* [57]. Periplasmic lysis was performed as described above, in lysis buffer (20 mM sodium phosphate pH 7, 150 mM NaCl, 20% w/v sucrose). Nickel affinity chromatography, using buffer A (20 mM sodium phosphate pH 7, 150 mM NaCl, 30 mM imidazole) and buffer B (20 mM sodium phosphate pH 7, 150 mM NaCl, 300 mM imidazole), and gel filtration using GF buffer (20 mM sodium phosphate pH 7, 150 mM NaCl) were conducted as for *Cj*X183. Pure protein was concentrated using a Vivaspin 10 000 Da cut off concentrator at 4000×***g*** and quantified using the A_280_ extinction coefficient of 53 775 M^−1^ cm^−1^ for *Cj*AA10BΔCBM and 81 275 M^−1^ cm^−1^ for *Cf*AA10.

### Crystallization, X-ray data collection and structure determination for *Cj*X183

*Cj*X183 was crystallized in 0.1 M HEPES pH 7.5, 20% w/v PEG 4000, 10% w/v propan-2-ol via sitting drop vapour-diffusion. Crystals were cryocooled by soaking crystals in mother liquor supplemented with 20% ethylene glycol for 30 s before plunging into liquid nitrogen. Diffraction data was collected at Diamond Light Source on beamline I03 with a wavelength of 0.976 Å. Data were indexed and integrated using XDS [77] with subsequent data processing performed in the CCP4i2 suite [78]. Anomalous diffraction from the heme iron atom was used for phasing using the SHELXC/D/E pipeline [79]. Model building and refinement was performed using iterative cycles of restrained refinement and model building with COOT [80] and REFMAC5 [81].

### Voltammetric electrochemical analysis of *Cj*X183

Cyclic voltammetry electrochemical measurements were performed using a standard three electrode set-up consisting of: a working pyrolytic graphite edge electrode attached to an Orgiatrod rotator operated in stationary mode; a saturated calomel reference electrode filled with saturated aqueous KCl solution, and a Pt wire counter electrode. For experiments on *Cj*X183 a 1 µl aliquot of protein was pipetted onto the surface of a freshly abraded working electrode and left to form an adsorbed film for ∼1 min. The three electrodes were contained within a custom-built electrochemical cell (constructed by the University of York Department of Chemistry Glass Workshop) surrounded by a thermostat-controlled water jacket which was maintained at 5°C. Calibration of the reference electrode to a standard hydrogen electrode gave a correction factor of +0.243 V which has been applied to the data. The measurements were performed in 50 mM sodium phosphate pH 7, 150 mM NaCl.

### UV-Visible detection of *Cj*X183 oxidation

Reduced *Cj*X183 was prepared by complete reduction using 100 mM ascorbate. Ascorbate was then removed by passing the sample through a PD-10 desalting column before *Cj*X183 was quantified using its A_552_ and the extinction coefficient for heme *c* of 27 500 M^−1^ cm^−1^. A batch of 20 µM *Cj*X183 was prepared, aliquoted and flash frozen in liquid N_2_. UV-Visible spectroscopy experiments were performed in 50 mM sodium phosphate pH 6 in 160 µl volumes in a quartz cuvette on a Cary60 spectrophotometer (Agilent). In the assay, reduced *Cj*X183 was used at 10 µM and LPMO concentrations were varied between 0 and 50 µM. Reactions were prepared by thawing aliquots of reduced *Cj*X183 and mixing 80 µl with 80 µl of LPMO at twice the desired concentration. Reactions were monitored at 552 nm with points taken every 0.1 s for 15 min. Data were fitted to a single exponential using the equation for one phase decay on GraphPad, from which rates of decay of the reduced state of *Cj*X183 could be extracted.

### Activity monitoring using matrix assisted laser desorption ionisation mass spectrometry

Activity assays were set up with phosphoric acid swollen cellulose (PASC). PASC was prepared as described by Wood [82]. Assays using chemically reduced *Cj*X183 were set up at room temperature with a volume of 1 ml in 5 mM ammonium acetate, pH 6, comprising 10% v/v PASC, 1 µM *Cj*AA10BΔCBM or *Cf*AA10 and 1 mM ascorbate or 150 µM reduced *Cj*X183. Assays were performed using either chemically reduced *Cj*X183 prepared as described above, or electrochemically reduced *Cj*X183. For electrochemical reduction, 2 ml of a 235 µM solution of *Cj*X183 in a pH 7 mixed buffer solution (consisting of 150 mM of NaCl and 50 mM each of acetate, Tris, phosphate and MES) was incubated at 0°C under an ambient atmosphere in the presence of 2.35 µM of the redox mediator methyl viologen and a carbon felt working electrode, held at −400 mV vs SHE for 1180 s. The oxidative state was monitored by the formation of a peak at 552 nm, indicative of the reduced state. Electrochemically reduced *Cj*X183 was then buffer exchanged using a Zeba spin desalting column into 50 mM sodium acetate pH 6 buffer before use. Activity assays for electrochemically reduced *Cj*X183 were set up in 50 mM sodium phosphate pH 6 with reaction constituents used at concentrations described above. Reactions were left for 16 h on the bench rotating head over tail at room temperature and analysed using Matrix Assisted Laser Desorption Ionisation Mass Spectrometry (MALDI-MS).

For MALDI-MS experiments, 1 µl of sample was mixed with an equivalent volume of 10 mg/ml 2,5-dihydroxybenzoic acid in 50% acetonitrile, 0.1% trifluoroacetic acid on a Bruker SCOUT-MTP 384 target plate. The spotted samples were then dried in air under a lamp before being analysed by mass spectrometry on a Ultraflex III matrix-assisted laser desorption ionization-time-of-flight/time-of-flight (MALDI-TOF/TOF) instrument (Bruker), as described in Hemsworth *et al.* [13].

### Activity monitoring using the Amplex Red H_2_O_2_ assay

Hydrogen peroxide production by *Cj*AA10BΔCBM and *Cf*AA10 was measured in 96 well plates on a FLUOstar Galaxy (BMG Labtech) plate reader using a coupled assay with Amplex Red [73]. Resorufin formation was detected in fluorescence mode using an excitation wavelength of 570 nm and an emission wavelength of 596 nm. All reactions were performed in triplicate in 50 mM sodium phosphate pH 6.0, at room temperature. H_2_O_2_ production comparison assays used ascorbate at 25 µM, *Cj*X183 at 50 µM and LPMOs at 2 µM. In assays used to determine the *K*_M_ and *V*_max_ for each enzyme, *Cj*AA10BΔCBM and *Cf*AA10 were used at 2 µM and concentration of reduced *Cj*X183 was varied around the *K*_M_ for each enzyme: between 0 and 64 µM for *Cj*AA10BΔCBM and 0–128 µM for *Cf*AA10 before settling on the final concentrations presented in Figure 4. 50 µM Amplex Red and 0.2 U ml^−1^ horseradish peroxidase were used for enzyme assays and assays were started with the addition of the electron donor. Reactions were monitored every minute for 120 min. The necessary LPMO free controls were performed alongside enzymatic assays, as *Cj*X183 and ascorbate will produce peroxide through oxidation in air. Fluorescence readings were converted to [H_2_O_2_] using a concentration curve performed with 0–10 µM H_2_O_2_ and measured at each concentration of *Cj*X183 to account for any inner filter effects that may arise from light absorption from the heme. Initial rates were measured using data from the first 10 min and fitted to a straight line, rates were plotted against substrate concentration and kinetic parameters were extracted using the Michaelis–Menten function on GraphPad.

### Glycoside hydrolase boosting activity assays

Boosting assays were set up in 500 µl volumes in 50 mM sodium phosphate pH 6 buffer with 10% v/v PASC or 10% w/v Avicel. Endo-1,4-β-d-Glucanase from *T. longibrachiatum* (Megazyme) was used at 1 U ml^−1^ (or ∼250 nM), *Cj*AA10BΔCBM and *Cf*AA10 were each used at 2 µM, with ascorbate used at 75 µM and *Cj*X183 at 150 µM. Reactions were incubated at room temperature, rotating head over tail for 8 h on PASC or 16 h on Avicel. Reactions were stopped by heating at 95°C for 10 min and the insoluble material was pelleted by centrifugation. Soluble material was diluted 10-fold into water, combined in a 1 : 1 ratio with 100 µM ^13^C labelled cellobiose (Omicron Biochemicals Inc.) and analysed by ESI-MS. Cellobiose production was quantified by comparing the intensity of the [cellobiose-Na]^+^ peak at 365 Da with that of the [^13^C cellobiose-Na]^+^ peak at 377 Da.

### Electrospray mass spectrometry to assess protein oxidation

Protein damage experiments were set up in 100 µl volumes in 50 mM sodium phosphate pH 6. 20 µM *Cj*AA10BΔCBM or *Cf*AA10 were mixed with 200 µM *Cj*X183 or 100 µM ascorbate. Samples were taken upon mixing, after 1 h, for the *Cf*AA10 enzyme, after 2 h, for the *Cj*AA10BΔCBM enzyme, and 24 h and analysed by electrospray mass spectrometry.

## Data Availability

The structure and accompanying structure factors for *Cj*X183 have been deposited with the protein data bank with accession code 7B21. All other data are present in the manuscript or supporting information. For access to raw data, please contact the corresponding author.

## Abbreviations

CDH: cellobiose dehydrogenase
ESI-MS: electrospray ionisation mass-spectrometry
FN3: fibronectin type III
LPMOs: lytic polysaccharide monooxygenases
MALDI-MS: Matrix Assisted Laser Desorption Ionisation Mass Spectrometry
PASC: phosphoric acid swollen cellulose

## Supplementary Material

Supplementary Tables S1-S3 and Figures S1-S3

Supplementary Spreadsheet S1
