# Supplementary material for: C-type cytochrome-initiated reduction of bacterial lytic polysaccharide monooxygenases
Source: Biochem J. 2021 Jul 28;478(14):2927–44. doi: 10.1042/BCJ20210376 (PMC8981238; doi:10.1042/BCJ20210376)
Supplement: Supplementary Tables S1-S3 and Figures S1-S3 [file BCJ-478-2927-s1.pdf]

Supporting Information for

Supplementary Notes

As described previously (1), reversible, non-catalytic electron transfer film voltammetry signals such as those recorded for *Cj*X183 (solid black line, Figure S1) can be analyzed by first fitting a polynomial baseline to the voltammogram (red line, Figure S1) and then subtracting this baseline to generate an "extracted signal" of the isolated Faradaic peak current, free from background non-Faradaic capacitive current contributions (dashed black line, Figure S1). A version of the Nernst equation (2) (Equation S1), can then be used to simulate the extracted signal.

$$i = \pm \frac{n_s n_{app} F^2 \nu A \Gamma}{RT} \frac{exp\{n_{app} F(E-E^0)/RT\}}{\left[1+exp\{n_{app} F(E-E^0)/RT\}\right]^2}$$ **Equation S1**

The known parameters in Equation S1 are F, the Faraday constant; ν, the experimental scan rate; A, the area of the electrode; R, the molar gas constant; T, temperature; and E, applied potential. Table S2 reports the parameters which have been chosen to generate the "simulated signal" data plotted in Figure S1. The "cathodic" values refer to those used to fit the reductive process (negative current peak), while "anodic" values refer to those used to fit the oxidative process (positive current peak). The values of n_s, the number of electrons in the reaction, and n_{app}, the apparent number of electrons, show that the experimental data for *Cj*X183 is well modelled by a one electron reaction. Averaging the two peak potential values (E^0 in the equation) yields the midpoint potential, $E^{f}_{(O/R)}$ / V vs SHE, as reported in main paper. The coverage of protein on the electrode, Γ, is consistent with a sub-monolayer coating of *Cj*X183.

Table S1. Cj*X183* Data collection and refinement statistics

	**CjX183**
**Data collection**	
Space group	P2 2_1 2_1
Cell dimensions	
a, *b*, *c* (Å)	28.51, 55.38, 55.86
α, β, γ (°)	90.0, 90.0, 90.0
Resolution (Å)	55.85-1.20 (1.22-1.20)
R_{merge}	0.05 (0.088)
R_{pim}	0.033 (0.057)
CC(1/2)	0.997 (0.990)
$I / \sigma I$	48.3 (21.7)
Completeness (%)	100 (99.6)
Redundancy	6 (6)
**Refinement**	
Resolution (Å)	39.36-1.20
No. reflections	28390/1404
R_{work} / R_{free}	0.112/0.128
B-factors ($Å^2$)	
Protein (1388 atoms)	11.8
Ligand (73 atoms)	6.8
Ion/solvent (19 atoms)	22.2
Water (117 atoms)	23.5
R.m.s. deviations	
Bond lengths (Å)	0.022
Bond angles (°)	1.945
PDB ID	7B21

*Values in parentheses are for highest-resolution shell.

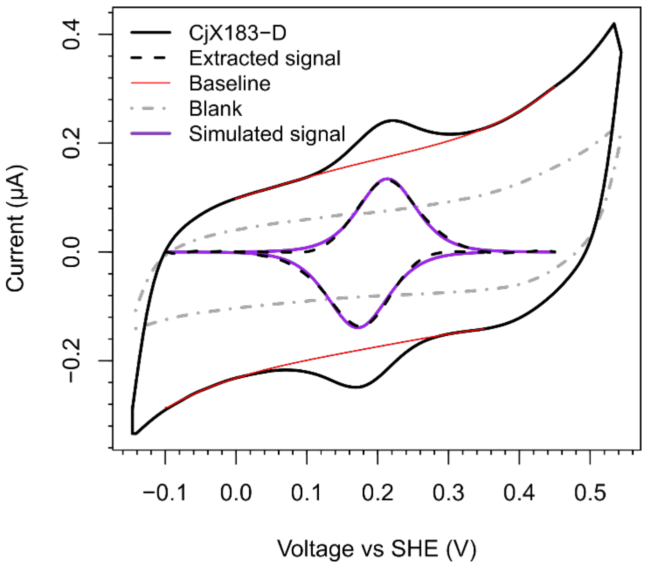

Figure S1. Cyclic voltammetry of CjX183. *30 mV s^{-1} cyclic voltammetry of a protein film of CjX183 adsorbed onto a pyrolytic graphite edge working electrode (solid black line) and the corresponding control "blank" experiment where a protein-free working electrode (gray dashed line) is measured under the same experimental conditions of pH 7.0 aqueous buffer (50 mM sodium phosphate, 150 mM NaCl), 5 °C, N_2 atmosphere, Pt counter electrode and calomel (saturated KCl) reference electrode (a correction factor has been applied to report potentials versus SHE). The extracted signal (black dashed line), baseline (solid red line) and simulated signal (solid purple line) relate to the analysis of the data.*

Table S2 Parameters used to generate the “simulated signal” data plotted in Figure S1.

**Parameter**	**Value**
A / cm^2	0.03
n_s cathodic	1
n_s anodic	1
n_{app} cathodic	0.835
n_{app} anodic	0.872
Anodic peak potential / mV vs SHE	214
Cathodic peak potential / mV vs SHE	171
$E^f_{(O/R)}$ / V vs SHE	193
Γ cathodic / pmol cm^{-2}	87
Γ anodic / pmol cm^{-2}	80

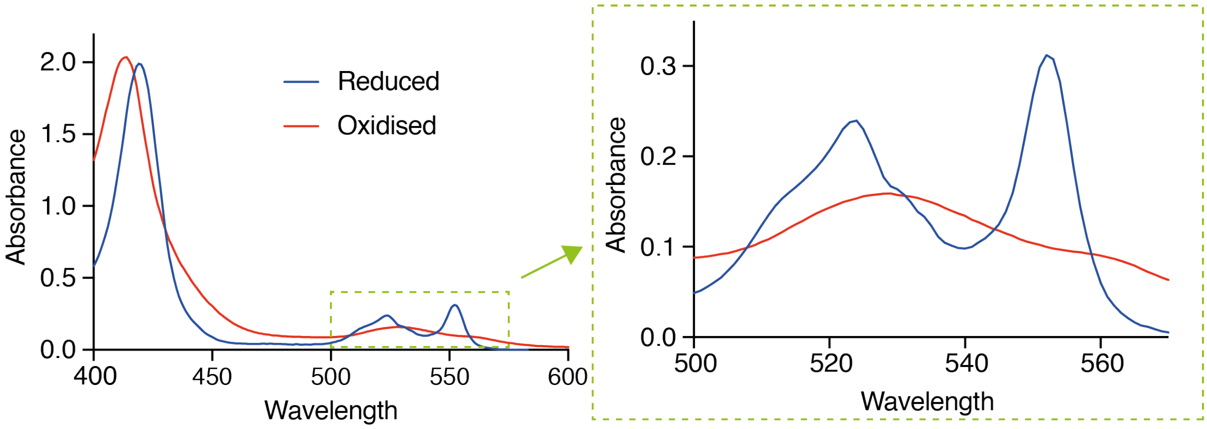

Figure S2: UV-Vis spectrum of CjX183 between 400 and 600nm. *The Soret band shifts from 413 nm to 419 nm upon reduction. In the 500-570 nm region, expanded in the right panel, the* α *and* β *bands, indicative of the reduced cytochrome, have absorption maxima at 523 nm and 552 nm, in the oxidised protein they form a broad peak.*

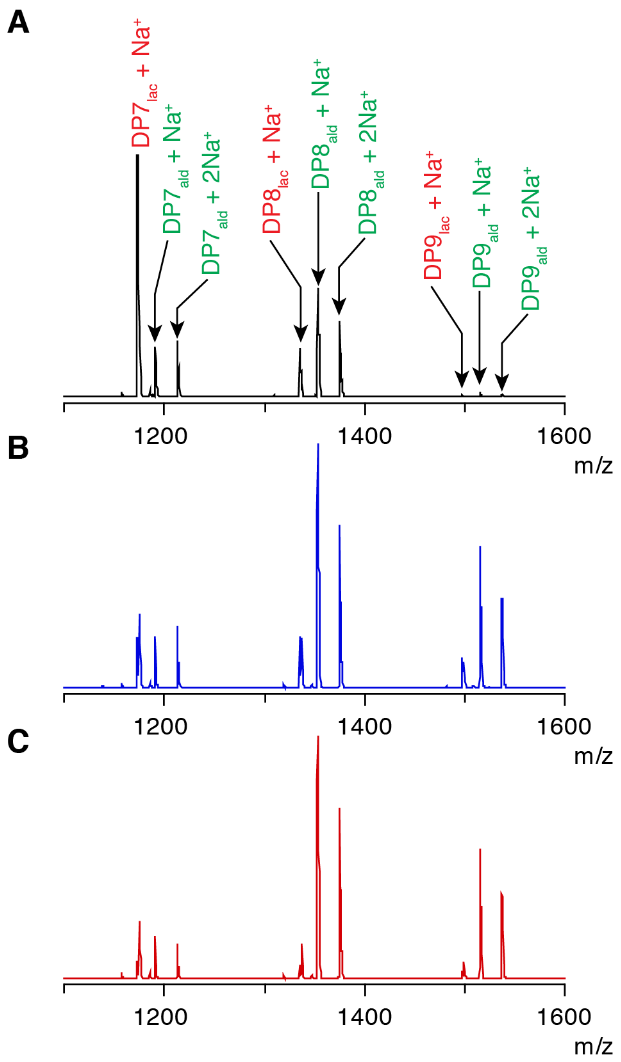

Figure S3. MALDI analysis of CfAA10 activity on PASC. *The oxidised oligosaccharide products liberated are shown for activity using* ***(A)*** *ascorbate as the reducing agent;* ***(B)*** *chemically reduced CjX183 as the activator; and* ***(C)*** *electrochemically reduced CjX183 as the activator.*

Table S3. Catalytic parameters calculated from the Amplex Red H_2O_2 assay.

	K_m (μM)	V_{max} (μM min^{-1})	k_{obs} (min^{-1})	K_{obs}/K_m (μM^{-1} min^{-1})
*Cj*AA10BΔCBM + *Cj*X183	0.78 ± 0.18	0.028 ± 0.002	0.014	0.018
*Cj*AA10BΔCBM + ascorbate	5.8 ± 0.3	0.049 ± 0.001	0.025	0.0042
*Cf*AA10 + *Cj*X183	5.7 ± 1.2	0.009 ± 0.006	0.004	0.00075
*Cj*AA10 + ascorbate	19.2 ± 1.2	0.111 ± 0.003	0.055	0.0029

Supplementary References

1. Yates, N. D., Dowsett, M. R., Bentley, P., Dickenson-Fogg, J. A., Pratt, A., Blanford, C. F., Fascione, M. A., and Parkin, A. (2020) Aldehyde-Mediated Protein-to-Surface Tethering via Controlled Diazonium Electrode Functionalization Using Protected Hydroxylamines. *Langmuir* **36**, 5654-5664

2. Heering, H. A., Weiner, J. H., and Armstrong, F. A. (1997) Direct Detection and Measurement of Electron Relays in a Multicentered Enzyme: Voltammetry of Electrode-Surface Films ofE. coliFumarate Reductase, an Iron−Sulfur Flavoprotein. *Journal of the American Chemical Society* **119**, 11628-11638
